# Activation of Akt/mTOR Pathway Is Associated with Poor Prognosis of Nasopharyngeal Carcinoma

**DOI:** 10.1371/journal.pone.0106098

**Published:** 2014-08-28

**Authors:** Weiyuan Wang, Qiuyuan Wen, Lina Xu, Guiyuan Xie, Jiao Li, Jiadi Luo, Shuzhou Chu, Lei Shi, Donghai Huang, Jinghe Li, Songqing Fan

**Affiliations:** 1 Department of Pathology, the Second Xiangya Hospital of Central South University, Changsha, Hunan, China; 2 Department of Oncology, the Second Xiangya Hospital of Central South University, Changsha, Hunan, China; 3 Department of Otorhinolaryngology, Xiangya Hospital of Central South University, Changsha, Hunan, China; 4 Department of Pathology, Basic Medical College of Central South University, Changsha, Hunan, China; The University of North Carolina at Chapel Hill, United States of America

## Abstract

Nasopharyngeal carcinoma (NPC) is a malignant tumor of the head and neck region, which frequently occurs in Southeast Asia, especially in the south of China. It is known that the mammalian target of rapamycin (mTOR) pathway plays a central role in regulating cellular functions, including proliferation, growth, survival, mobility, and angiogenesis. Aberrant expression of the mTOR signaling pathway molecules has been found in many types of cancer. However, whether the alterations of p-Akt, p-p70S6K and p-4EBP1 protein expression are associated with clinicopathological features and prognostic implications in NPC have not been reported. The purposes of the present study are to investigate the association between the expression of p-Akt, p-p70S6K and p-4EBP1 proteins and clinicopathological features in NPC by immunohistochemistry. The results showed that the positive percentage of p-Akt, p-p70S6K and p-4EBP1 proteins expression in NPC (47.2%, 73.0% and 61.7%, respectively) was significantly higher than that in the non-cancerous nasopharyngeal control tissue (33.3%, 59.1% and 47.0%, respectively). There was a significantly higher positive expression of p-Akt in undifferentiated non-keratinizing nasopharyngeal carcinoma than that in differentiated non-keratinizing nasopharyngeal carcinoma (*P* = 0.014). Additionally, positive expression of p-p70S6K and p-4EBP1 proteins, and positive expression of either of p-Akt, p-p70S6K and p-4EBP1 were significantly correlated inversely with overall survival rates of NPC patients (*P* = 0.023, *P* = 0.033, *P* = 0.008, respectively). Spearman’s rank correlation test showed that expression of p-Akt in NPC was significantly associated with expression of p-p70S6K (r = 0.263, *P*<0.001) and p-4EBP1(r = 0.284, *P*<0.001). Also there was an obviously positive association between expression of p-p70S6K and p-4EBP1 proteins in NPC (r = 0.286, *P*<0.001). Multivariate Cox regression analysis further identified positive expression of p-4EBP1 and p-p70S6K proteins were the independent poor prognostic factors for NPC (*P* = 0.043, *P* = 0.027, respectively). Taken together, high expression of p-p70S6K and p-4EBP1 proteins may act as valuable independent biomarkers to predict a poor prognosis of NPC.

## Introduction

Nasopharyngeal carcinoma (NPC) is a kind of head and neck malignant tumor, which frequently occurs in Southeast Asia, especially in the south of China [1]. It appears that multiple factors are involved in the carcinogenesis of NPC ranging from Epstein-Barr virus (EBV) infection, environmental factors and genetic susceptibility, to the aberrant expression of oncogenes/tumor-suppressor genes and the abnormal activation of signaling pathways such as Akt/mTOR pathway, mitogen-activated protein kinases and Wnt signaling pathway [1]–[2]. Radiation therapy is the major therapeutic modality used to treat NPC, and most NPC patients can be cured if the disease is diagnosed and treated at an early stage, whereas, in advanced stages, local recurrence and metastasis are still the leading cause of mortality [3]. Therefore, further elucidation of the molecular mechanism and novel targets underlying NPC is vital for the development of new effective therapeutic agents.

Akt, a serine–threonine kinase, plays a central role in the PI3K/Akt/mTOR signaling pathway. Consecutively, phosphorylation of mTOR regulates two downstream effectors named p70S6K (p70 ribosomal protein S6 kinase) and 4E-BP1 (eukaryotic initiation factor 4E-binding protein1) [4]–[5]. The eIF4E is released to initiate translation after 4E-BP1 phosphorylation, while p70S6K translates mRNA transcripts in the form of 5′TOP motif next to the phosphorylation by mTOR [6]–[7]. Activations of 4E-BP1 and p70S6K can control protein synthesis and act as proto-oncogenes [8]–[9]. It is known that the Akt/mTOR signaling pathway plays a central role in regulating cellular functions, including proliferation, growth, survival, mobility, and angiogenesis [10]–[11]. Aberrant expression of Akt/mTOR signaling pathway molecules has been seen in many types of cancer, such as non-small-cell lung cancer, colorectal cancer, and liver cancer [12]–[14]. Akt, p70S6K and 4EBP1 are three key proteins of Akt/mTOR signaling pathway. However whether expression of p-Akt, p-p70S6K and p-4EBP1 proteins is associated with clinicopathological features and prognostic implications in NPC has not been reported.

In this study, we detected phosphorylated forms of the three key constituent proteins on the Akt/mTOR signaling pathway (p-Akt, p-p70S6K and p-4E-BP1) by immunohistochemistry (IHC) in 248 cases of NPC and 66 cases of non-cancerous nasopharyngeal control tissue, and investigated the correlations between the expression of p-Akt, p-p70S6K and p-4EBP1 proteins and clinicopathological features and prognostic implications in NPC. We found that positive percentage of p-Akt, p-p70S6K and p-4EBP1 expression in NPC was significantly higher than that in the non-cancerous nasopharyngeal control tissue. Expression of p-4EBP1 and p-p70S6K proteins was identified as the independent poor prognostic factors for NPC.

## Materials and Methods

### Ethics Statement

Samples were obtained with informed consent and all protocols were approved by the Second Xiangya Hospital of Central South University Ethics Review Board (Scientific and Research Ethics Committee, no. s02/2000). Written informed consent was obtained from all patients also the written informed consent was obtained from the next of kin, caretakers, or guardians on the behalf of the minors/children participants involved in your study.

### Tissue samples and clinical data

Two hundred and forty-eight (248) cases of paraffin-embedded NPC from the primary NPC patients with their age ranging from 17 to 73 years (median, 46.8 years), also 66 cases of non-cancerous nasopharyngeal control specimen from independent patients with chronic inflammation of nasopharyngeal mucosa, were obtained from the Second Xiangya Hospital of Central South University (Changsha, China). No patient had previously been treated with radiotherapy and chemotherapy at the time of original biopsy. Complete clinical record and follow-up data were available for all patients. Also, written informed consent was obtained from all patients. This study protocol, specimen usage, and data retrieval were approved by the Institutional Human Experiment and Ethics Committee of the Second Xiangya Hospital of Central South University (approval number S-02-2000). All specimens had been confirmed pathological diagnosis according to the 2005 WHO histological classification of NPC. The patients were staged according to the UICC/AJCC 1997 staging system of NPC.

Histological patterns and clinical stages of NPC were classified as follows: 223 cases of undifferentiated non-keratinizing nasopharyngeal carcinoma and 25 cases of differentiated non-keratinizing nasopharyngeal carcinoma; 9 cases of clinical stage I, 86 cases of stage II, 94 cases of stage III, and 59 cases of stage IV. Among these patients included in the study, 166 patients were positive for cervical lymph node metastasis and 82 patients were negative. Among 248 patients, 130 patients were treated by radiotherapy alone, 9 patients by chemotherapy alone, and 109 patients by combined radiotherapy and chemotherapy. Complete clinical record and follow-up data of all patients were available. Overall survival time was calculated from the data of diagnosis to the date of death or the data last known alive. A total of 157 patients (63.3%) were alive with a mean follow-up period of 44.6 months (10–120 months).

### IHC and Scores

The IHC staining for p-Akt, p-4EBP1 and p-p70S6K proteins in NPC sections was carried out using ready-to-use Envision^+^Dual Link System-HRP methods (Dako; Carpinteria, CA). The staining conditions for each antibody were adjusted according to our laboratory experience. Briefly, each section was deparaffinized and rehydrated, and high-temperature antigen retrieval was achieved for all antibodies by heating the samples in 0.01 M citrate buffer in a domestic microwave oven at full power (750 Watts) for 15 minutes, then the samples were immersed into methanol containing 0.3% H_2_O_2_ to inactivate endogenous peroxidase at 37°C for 30 minutes. To eliminate nonspecific staining, the slides were incubated with appropriate preimmune serum for 30 minutes at room temperature. After incubation with a 1∶100 dilution of primary antibody to p-Akt (pS473) protein (Rabbit polyclonal, Catalog:#2227-1, Epitomics, Inc.) and with a 1∶500 dilution of primary antibody to p-4EBP1 protein (Rabbit polyclonal IgG p-4EBP1(Thr37/46) (Catalog:#2855, Cell Signaling) and with a 1∶500 dilution of primary antibody to p-p70S6K (Thr389) protein (Catalog: #9206, Cell Signaling) at 4°C overnight, slides washed with physiological phosphate buffered saline (PBS) three times for five minutes each and second antibody conjugated with a labeled polymer-HRP was added according to the manufacturer’s instructions and incubated at 37°C for 30 minutes. Color reaction was developed by using 3-amino-9-ethylcarbazole (AEC) chromogen solution. All slides were counterstained with hematoxylin. Positive control slides were included in every experiment in addition to the internal positive control. The specificity of the antibody was determined with matched IgG isotype antibody as a negative control.

Immunohistochemical staining was evaluated independently by QW and LX who were blinded to the clinicopathological data, at 200× magnification light microscopy. Positive expression of p-Akt protein was identified in cells membrane, positive expressions of p-4EBP1 and p-p70S6K were found in the cytoplasm. A semi-quantitative evaluation of p-Akt, p-4EBP1 and p-p70S6K was performed using a method described in the literature [30] as follows: the percentage of positive cells was divided into five grades (percentage scores): ≤10% (0), 11–25% (1), 26–50% (2), 51–75% (3), and >75% (4). The intensity of staining was divided into four grades (intensity scores): no staining (0), light brown (1), brown (2), and dark brown (3). Staining positivity was determined by the formula: overall scores = percentage score×intensity score. The total score ranged from 0 to 12, with negative staining (0–1) and positive expression (2–12). Agreement between the two evaluators was 95%, and all scoring discrepancies were resolved through discussion between the two evaluators.

### Statistical analyses

All statistical analyses were performed using SPSS 13.0. The chi-square test was used to analyze the relationship between the expression of p-Akt, p-4EBP1 and p-p70S6K proteins and clinicopathological characteristics and prognostic factors in NPC. The Spearman’s rank correlation coefficient was used to assess the significance of the association among expression of p-Akt, p-4EBP1 and p-p70S6K proteins in NPC. Kaplan-Meier analysis was performed for overall survival curves and statistical significance was assessed using the log-rank test. Overall survival was defined as the time from the treatment initiation (diagnosis) to the date of death. To evaluate whether expression of p-Akt, p-4EBP1 and p-p70S6K proteins are the independent prognostic factors of overall survival for NPC patients, multivariate analysis using the Cox proportional hazard regression model was performed. All *p-* values were based on the two-sided statistical analysis and *p*-value less than 0.05 was considered to be statistically significant.

## Results

### Association between expression of p-Akt, p-4EBP1 and p-p70S6K proteins and clinicopathological features of NPC

**Figure 1:**
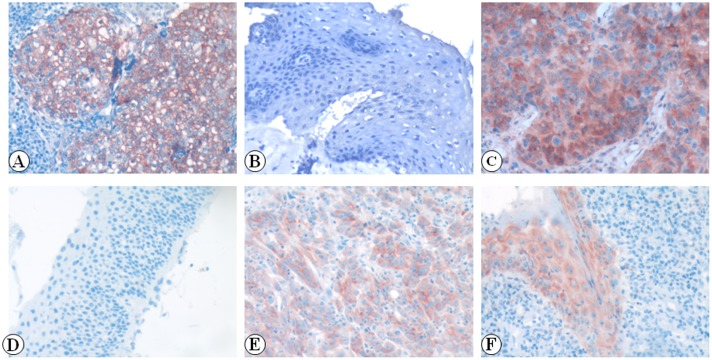
Expression of p-Akt, p-4EBP1 and p-p70S6K proteins in NPC and non-cancerous nasopharyngeal control tissue was detected by IHC. The expression of p-Akt, p-4EBP1 and p-p70S6K proteins were detected by IHC using specific antibodies as described in the section of materials and methods. Strong expression of p-Akt protein was identified in NPC cells membrane (Fig. 1A, 20×, IHC, AEC staining). Negative staining of p-Akt protein was identified in the control nasopharyngeal epithelia (Fig. 1D, 20×, IHC, AEC staining). Strong staining of p-4EBP1 was found in the cytoplasm of NPC (Fig. 1B, 20×, IHC, AEC staining). Negative staining of p-4EBP1 was in the control nasopharyngeal epithelia (Fig. 1E, 20×, IHC, AEC staining). Strong staining of p-p70S6K was found in the cytoplasm of NPC (Fig. 1C, 20×, IHC, AEC staining). Moderate staining of p-p70S6K was in cytoplasm of control nasopharyngeal epithelial cells (Fig. 1F, 20×, IHC, AEC staining).

We examined the positive expression and cellular location of p-Akt, p-4EBP1 and p-p70S6K in NPC and non-cancerous nasopharyngeal control tissue by IHC. Strong expression of p-Akt protein was identified on the NPC cell membranes (Fig. 1A) and negative staining of p-Akt was identified in the control nasopharyngeal epithelial cells (Fig. 1B). Strong staining of p-4EBP1 was found in the cytoplasm of NPC (Fig. 1C) and negative staining of p-4EBP1 protein was in the control nasopharyngeal epithelial cells (Fig. 1D). Strong staining of p-p70S6K was found in the cytoplasm of NPC (Fig. 1E) and moderate staining of p-p70S6K was showed in the cytoplasm of control nasopharyngeal epithelial cells (Fig. 1F). The positive percentage of p-Akt, p-4EBP1 and p-p70S6K expression in the NPC and the non-cancerous nasopharyngeal control tissue was 47.2% (117/248), 73.0% (181/248), 61.7% (153/248), 33.3% (22/66), 59.1% (39/66) and 47.0% (31/66), respectively. There were significantly higher expression of p-Akt, p-4EBP1 and p-p70S6K proteins in NPC compared with the non-cancerous nasopharyngeal control tissue (*P* = 0.044, *P* = 0.029, *P* = 0.031, respectively).

We further investigated the associations between the expression of p-Akt, p-4EBP1 and p-p70S6K proteins and clinicopathological features of NPC including age, gender, histological type, clinical stage, lymph node metastasis status and survival status in univariate chi-square test. A comparison of undifferentiated non-keratinizing nasopharyngeal carcinoma and differentiated non-keratinizing nasopharyngeal carcinoma showed that significantly higher positive expression of p-Akt (*P* = 0.014) was found in undifferentiated non-keratinizing nasopharyngeal carcinoma. However, no significant differences were observed between expression of p-4EBP1 and p-p70S6K proteins and histological type of NPC. Data showed in table 1 also indicated a significantly negative correlation between expression of p-4EBP1 (*P* = 0.024) and p-p70S6K (*P* = 0.033) proteins and the survival status of NPC patients. A conjoint analysis indicated that positive expression of either of p-Akt, p-4EBP1 and p-p70S6K proteins had an evidently inverse correlation with survival status of NPC patients (*P*<0.01). However, there was no significant correlation observed between expression of p-Akt and overall survival rates of NPC patients. Also, no correlation was discovered between positive expression of either of three proteins above and age, gender, clinical stages and lymph node metastasis of NPC (*P*>0.05, respectively).

**Table 1:** Association between expression of p-Akt, p-p70S6K and p-4EBP1 and NPC clinical pathological features (N = 248).

Characteristics (N)	p-Akt			p-4EBP1			p-p70S6K			p-Akt/p-4EBP1/p-p70S6K*		
	P (%)	N (%)	*P-value*	P (%)	N (%)	*P-value*	P (%)	N (%)	*P-value*	P^+^ (%)	N^−^ (%)	*P-value*
**Age(yr)**												
≤40(n = 82)	42(51.2)	40(48.8)	0.370	61(74.4)	21(25.6)	0.726	49 (59.8)	33 (40.2)	0.659	68 (82.9)	14 (17.1)	0.682
>40 (n = 166)	75(45.2)	91(54.8)		120(72.3)	46(27.7)		104(62.7)	62(37.3)		141(84.9)	25 (15.1)	
**Gender**												
Female (n = 61)	35 (57.4)	26(42.6)	0.066	47 (77.0)	14 (23.0)	0.410	36(59.0)	25(41.0)	0.620	53 (86.9)	8 (13.1)	0.410
Male (n = 187)	82 (43.9)	105(56.1)		134(71.7)	53 (28.3)		117(62.6)	70 (37.4)		156(83.4)	31(16.6)	
**Histological type**												
DNC (n = 25)	6 (24.0)	19 (76.0)	0.014	15 (60.0)	10 (40.0)	0.123	15 (60.0)	10 (40.0)	0.854	20 (80.0)	5(20.0)	0.536
UDNC (n = 223)	111(49.8)	112(50.2)		166(74.4)	57 (25.6)		138(61.9)	85 (38.1)		189(84.8)	34 (15.2)	
**Clinical stages**												
Stages _I-II_ (n = 95)	43 (45.3)	52 (54.7)	0.634	69 (72.6)	26 (27.4)	0.922	61 (64.2)	34(35.8)	0.521	78 (82.1)	17(17.9)	0.460
Stages _III-IV_ (n = 153)	74 (48.4)	79 (51.6)		112(73.2)	41 (26.8)		92 (60.1)	61(39.9)		131(85.6)	22 (14.4)	
**LN status**												
LNM (n = 166)	76(45.8)	90(54.2)	0.531	126(75.9)	40 (24.1)	0.141	105(63.3)	61(36.7)	0.472	144(86.7)	22 (13.3)	0.128
No LNM (n = 82)	41 (50.0)	41(50.0)		55(67.1)	27(32.9)		48 (58.5)	34 (41.5)		65 (79.3)	17(20.7)	
**Survival status**												
Alive (n = 157)	72 (45.9)	85 (54.1)	0.585	107(68.2)	50 (31.8)	0.024	89 (56.7)	68 (43.3)	0.033	125(79.6)	32 (20.4)	0.008
Dead (n = 91)	45 (49.5)	46(50.5)		74 (81.3)	17(18.7)		64 (70.3)	27(29.7)		84 (92.3)	7 (7.7)	

**Abbreviations:** DNKC: Differentiated non-keratinized nasopharyngeal carcinoma; UDNC: Undifferentiated non-keratinized nasopharyngeal carcinoma; LN: lymph node; LNM, lymph node metastasis; P: positive; N: negative.

*:P^+^: either of positive expression of p-Akt, p-p70S6K and p-4EBP1; N^−^: common negative staining of three proteins.

### The pairwise association between expression of p-Akt, p-4EBP1 and p-p70S6K proteins in NPC

The pairwise association between aberrant expression of p-Akt, p-4EBP1 and p-p70S6K proteins in NPC was revealed in table 2. Positive expression of p-Akt were significantly associated with expression of p-p70S6K (r = 0.263, *P*<0.001) and p-4EBP1 (r = 0.284, *P*<0.001) proteins in NPC. There was also significantly positive association between expression of p-p70S6K and p-4EBP1 proteins in the NPC (r = 0.286, *P*<0.001).

**Table 2:** The pairwise association between expression of p-Akt, p-p70S6K and p-4EBP1 protein in the 248 cases of NPC.

	p-AKt	p-p70S6K	p-4EBP1
**p-AKt**			
Spearman’s Correlation Coefficient Sig. (2-tailed)	1	0.263**	0.284**
**p-p70S6K**			
Spearman’s Correlation Coefficient Sig. (2-tailed)	–	1	0.286**

NOTE. Values are Spearman’s correlation coefficient, ** Correlation is significant at the p<0.01 level (2-tailed).

### Impact of expression of p-Akt, p-4EBP1 and p-p70S6K proteins on the prognosis of NPC patients

**Figure 2:**
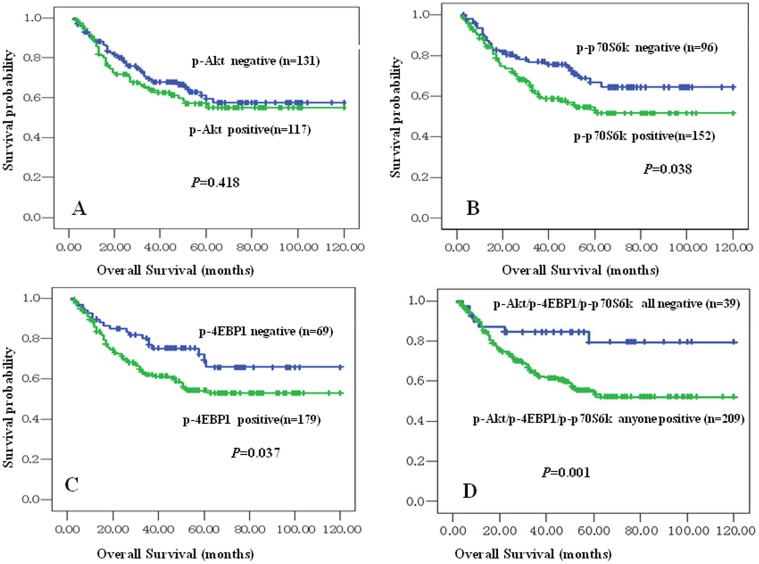
Kaplan-Meier overall survival curves of NPC patients with expression of p-Akt, p-4EBP1 and p-p70S6K proteins and different clinicopathological characteristics. Kaplan-Meier analysis to plot the survival curve of 248 cases of NPC with expression of p-Akt, p-4EBP1 and p-p70S6K and clinicopathological characteristics and statistical significance were assessed using the log-rank test. Fig. 2A: Positive expression of p-Akt had no significantly correlation with overall survival rates of NPC patients (*P*>0.05, two sided). Fig. 2B: Kaplan-Meier curves showed worse overall survival rates for NPC patients with positive expression of p-p70S6K protein compared to patients with p-p70S6K negative staining (*P* = 0.038, two sided). Fig. 2C: Kaplan-Meier curves showed worse overall survival rates for NPC patients with positive expression of p-4EBP1 protein compared to patients with p-4EBP1 negative staining (*P* = 0.037, two sided). Fig. 2D: Kaplan-Meier curves showed worse overall survival rates for NPC patients with either of p-Akt, p-p70S6K and p-4EBP1 positive expression compared to patients with common negative staining of three proteins above (*P* = 0.001, two sided).

In univariate survival analysis of NPC patients, Kaplan-Meier survival curve analysis with log-rank significance test was performed. Figure 2 illustrated the Kaplan-Meier survival plots for NPC patients with different expression of p-Akt (Fig. 2A), p-p70S6K (Fig. 2B), p-4EBP1 (Fig. 2C) and combination of expression of either of three proteins above (Fig. 2D). The overall survival rates for NPC patients with negative expression of p-p70S6K protein was slightly higher than these with positive p-p70S6K expression (*P* = 0.038, Fig. 2B), as well as the overall survival rates for NPC patients with negative expression of p-4EBP1 was better than these with positive p-4EBP1 expression (*P* = 0.037, Fig. 2C). In addition, those patients with positive expression with either of p-Akt, p-4EBP1 and p-p70S6K proteins might have a lower survival probability than patients with all negative staining of three proteins above (*P* = 0.001, Fig. 2D). However, there was no significant association between expression of p-Akt and overall survival rates in NPC patients (*P*>0.05).

Besides univariate analysis, multivariate Cox proportional hazard regression analysis was also carried out to further investigate whether the expression of p-Akt, p-p70S6K, p-4EBP1 proteins are the independent prognostic factors of NPC. These results were revealed in table 3. In multivariate analysis of 248 cases of NPC, which included clinical stages, lymph node metastasis status, histological type, treatment strategy, age and gender, expression of p-Akt, p-p70S6K and p-4EBP1 proteins, results showed that positive expression of p-4EBP1 and p-p70S6K proteins were identified as the independent poor prognostic factors for NPC (*P* = 0.043, *P* = 0.027, respectively), as did clinical stages (*P*<0.001) and treatment strategy (*P* = 0.007). Again, no impact was detected with age, gender and histological type of NPC (*P*>0.05 for all).

**Table 3:** Summary of multivariate analysis of Cox proportional hazard regression for overall survival in 248 cases of NPC.

Parameter	Wald	Sig.	Exp(B)	95.0% CI for Exp(B)	
				Lower	Upper
**p-Akt**	.007	.935	.982	.636	1.517
**p-p70S6K**	4.086	.043*	1.631	1.015	2.622
**p-4EBP1**	4.890	.027*	1.893	1.075	3.334
**clinical stages**	26.243	.000*	2.076	1.570	2.746
**LNM status**	1.222	.269	1.337	.799	2.238
**Histological type**	.365	.546	.803	.394	1.637
**Treatment strategy**	7.273	.007*	.540	.346	.845
**Age**	1.792	.181	1.384	.860	2.226
**Gender**	2.179	.140	.665	.387	1.143

**Abbreviations:** LNM, lymph node metastasis; CI, confidence interval.

**Note:** multivariate analysis of Cox regression,*:p<0.05.

## Discussion

Previous study has shown that Akt/mTOR signaling pathway plays an important role in the execution of numerous cellular processes including cell growth, proliferation, cell differentiation and survival [10]–[11]. Akt is an important kinase of Akt/mTOR signaling pathway which could phosphorylate the p70S6K and 4EBP1, and both of p70S6K and 4EBP1 are major downstream targets of Akt/mTOR signaling pathway which could regulate protein synthesis and cell cycle progression [14]–[17]. Phosphorylated Akt is detectable and associated with a poor prognosis in multiple human cancers, such as non-small cell lung cancer, breast cancer and acute lymphoblastic leukemia [12], [18]–[19]. Akt promotes chemoresistance in human ovarian cancer cells by modulating cisplatin-induced, p53-dependent ubiquitination of FLICE-like inhibitory protein [20]. Another study has discovered that radiation is able to induce Akt activity which could modulate radioresistance in human glioblastoma cells [21]. And these further indicate the important role of activation of Akt in tumor progression. In our present study, results showed that p-Akt was significantly increased in NPC compared with non-cancerous nasopharyngeal control tissue, so it suggested that p-Akt might play an important role in promoting the development and progression of NPC. Also, there was a significantly higher positive expression of p-Akt in the undifferentiated non-keratinizing nasopharyngeal carcinoma than that in differentiated non-keratinizing nasopharyngeal carcinoma. However, there was no significant correlation observed between expression of p-Akt and overall survival rates of NPC patients. Our result was different from other solid tumors previously reported. For our consideration, Akt could activate many downstream factors and participate in regulation of downstream pathways. Therefore, its role should be comprehensively evaluated. Besides, genetic and environmental factors, like Epstein-Barr virus (EBV), cause various influences in different histological types of NPC [22]. Obviously, further studies will be also required to elucidate the precise mechanism of the role of p-Akt protein in NPC.

As far as 4EBP1 and p70S6K are concerned, 4EBP1 takes part in controlling ribosome protein synthesis,and therefore, non-phosphorylated 4E-BP1 binds eIF4E and impedes formation of the initiation complex, blocking translation, favoring apoptosis and inhibiting tumor cell survival and proliferation. Phosphorylation of 4EBP1 results in the release of eIF4E and activates protein synthesis [23]. Recent study has shown that overexpression of p-4EBP1 in breast cancer indicates poor prognosis [24]. The p70S6K is a kind of cytoplasmic serine/threonine kinase that regulates protein translation mainly through phosphorylation of the 40S ribosomal protein S6 [25]. Aberrant expression of p-p70S6K possibly contributes to pathogenesis, growth, invasion and metastasis of gastric carcinomas [26]. Our results indicated that expression of p-4EBP1 and p-p70S6K proteins in NPC was significantly higher than that in the non-cancerous nasopharyngeal control tissue. There was a significant negative association between positive expression of p-4EBP1 and p-p70S6K proteins and survival status of NPC patients. Interestingly, in our study, it is noteworthy that those NPC patients with positive expression with either of p-Akt, p-4EBP1 and p-p70S6K proteins might have a lower survival probability than patients with all negative staining of three proteins above. Our results suggest that high expression of p-Akt, p-4EBP1 and p-p70S6K proteins may participate in promoting cell survival and proliferation and associate with the poor prognosis of NPC patients. In addition, positive expression of p-Akt was significantly associated with expression of p-p70S6K and p-4EBP1. There was also a significant positive association between expression of p-p70S6K and p-4EBP1 proteins in NPC. These findings indicated that increased expression of Akt/mTOR signaling pathway could play an important role in the pathogenesis of NPC.

Abnormal expression of the Akt/mTOR signaling pathway has been observed in various solid tumors [24], [27]–[29]. Our results showed that the NPC patients with positive expression of p-4EBP1 and p-p70S6K proteins had an obviously shorter survival time than these patients with negative staining of p-4EBP1 and p-p70S6K. Furthermore, multivariate analysis proved that the positive expression of p-4EBP1 and p-p70S6K proteins were the independent factors of poor prognosis in NPC regardless of clinical stages, histological type, age and gender. Therefore, aberrant expression of p-4EBP1 and p-p70S6K acts as novel prognostic markers for NPC.

In summary, we first reported that the high expression of p-4EBP1 and p-p70S6K and positive expression of either of p-Akt, p-4EBP1 and p-p70S6K proteins were associated with the poor overall survival rates of NPC. Furthermore, multivariate analysis indicated that positive expression of p-4EBP1 and p-p70S6K proteins might be regarded as the independent prognostic factors for poor prognosis in NPC patients.
